# Organophosphate-Induced Changes in the PKA Regulatory Function of Swiss Cheese/NTE Lead to Behavioral Deficits and Neurodegeneration

**DOI:** 10.1371/journal.pone.0087526

**Published:** 2014-02-18

**Authors:** Jill S. Wentzell, Marlène Cassar, Doris Kretzschmar

**Affiliations:** Center for Research on Occupational and Environmental Toxicology, Oregon Health & Sciences University, Portland, Oregon, United States of America; National Center for Geriatrics and Gerontology, Japan

## Abstract

Organophosphate-induced delayed neuropathy (OPIDN) is a Wallerian-type axonopathy that occurs weeks after exposure to certain organophosphates (OPs). OPs have been shown to bind to Neuropathy Target Esterase (NTE), thereby inhibiting its enzymatic activity. However, only OPs that also induce the so-called aging reaction cause OPIDN. This reaction results in the release and possible transfer of a side group from the bound OP to NTE and it has been suggested that this induces an unknown toxic function of NTE. To further investigate the mechanisms of aging OPs, we used *Drosophila*, which expresses a functionally conserved orthologue of NTE named Swiss Cheese (SWS). Treating flies with the organophosporous compound tri-ortho-cresyl phosphate (TOCP) resulted in behavioral deficits and neurodegeneration two weeks after exposure, symptoms similar to the delayed effects observed in other models. In addition, we found that primary neurons showed signs of axonal degeneration within an hour after treatment. Surprisingly, increasing the levels of SWS, and thereby its enzymatic activity after exposure, did not ameliorate these phenotypes. In contrast, reducing SWS levels protected from TOCP-induced degeneration and behavioral deficits but did not affect the axonopathy observed in cell culture. Besides its enzymatic activity as a phospholipase, SWS also acts as regulatory PKA subunit, binding and inhibiting the C3 catalytic subunit. Measuring PKA activity in TOCP treated flies revealed a significant decrease that was also confirmed in treated rat hippocampal neurons. Flies expressing additional PKA-C3 were protected from the behavioral and degenerative phenotypes caused by TOCP exposure whereas primary neurons were not. In addition, knocking-down PKA-C3 caused similar behavioral and degenerative phenotypes as TOCP treatment. We therefore propose a model in which OP-modified SWS cannot release PKA-C3 and that the resulting loss of PKA-C3 activity plays a crucial role in developing the delayed symptoms of OPIDN but not in the acute toxicity.

## Introduction

Organophosphate-induced delayed neuropathy (OPIDN) is a degenerative syndrome that results from exposure to organophosphates (OPs) found in pesticides and nerve agents. OPIDN is characterized by a “dying-back” axonopathy leading to progressive limb weakness, abnormal reflexes, and paralysis which typically occur weeks or even months after exposure [1]–[3]. This syndrome was first described in 1930 after a poisoning epidemic in the southern United States, when thousands of people were paralyzed after consuming a beverage called Jamaica Ginger that contained the organophosphorus compound tri-ortho-cresyl phosphate (TOCP) [4]. The primary molecular target connected with the development of OPIDN is a protein called neuropathy target esterase (NTE). NTE has been shown to act as a phospholipase [5]–[8], a function that is mediated by a conserved domain that includes the active site serine [9]. This serine also provides the binding site for OPs [9], resulting in an inhibition of the enzymatic activity of NTE [10]. However, a number of observations have suggested that the mechanism of OP toxicity might be more complex. To induce OPIDN in chickens, NTE esterase activity must be at least 70% inhibited [7], [11]–[13]; however, some OPs can inhibit the activity by more than 80%, but do not induce OPIDN [14]. In contrast to the neuropathic, OPIDN-inducing OPs, these non-neuropathic OPs fail to induce the so-called “*aging reaction*”, in which a side group is released from the bound OP, resulting in a negative charge at the active site and, in the case of at least some OPs, a transfer of the released side group within NTE [15], [16]. It has therefore been proposed that the aging reaction affects a non-esterase function of NTE, inducing a novel toxic function that is required for developing the full symptoms of OPIDN [11], [12], [16]–[18].

The *Drosophila* orthologue of NTE is Swiss Cheese (SWS), which shows 39% overall identity to human and mouse NTE and 61% identity in the phospholipase domain, including the active site serine [9]. Like NTE, SWS shows phospholipase activity and their functional conservation was further confirmed by the result that mouse NTE can completely replace SWS in *sws* mutant flies (Muhlig-Versen et al., 2005). *Swiss cheese (sws)* mutant flies show age-dependent neurodegeneration, glial hyperwrapping, and neuronal apoptosis [19]. Mice lacking NTE show severe growth retardation and die around day 9 of embryonic development [20], whereas heterozygotes are hyperactive and show increased lethality after OP treatment [7]. In contrast, brain specific NTE knock-out mice survive into adulthood and show a strikingly similar phenotype to *sws* mutants, including vacuolization, abnormal myelin figures, and neuronal death [21]. Mutations in human NTE cause a Hereditary Spastic Paraplegia, now called NTE-related Motor-Neuron Disorder (NTE-MND), that is characterized by progressive spastic weakness starting in childhood [22]. Although these mutations have been shown to reduce the enzymatic activity of NTE in these patients, a similar reduction was found in asymptomatic subjects that are heterozygous for an insertion that deletes the phospholipase domain [23]. This suggests that, as for OPIDN, a reduction in the enzymatic activity alone may not be sufficient for developing NTE-MND.

We recently showed, that in addition to the phospholipase activity, SWS can also function as a non-canonical regulatory subunit of Protein Kinase A (PKA). SWS binds and inhibits specifically the C3 catalytic subunit (PKA-C3) which together with orthologues in mouse (Pkare) and human (PrKX) forms a novel class of catalytic subunits of unknown function (Bettencourt da Cruz et al., 2008). We have now investigated a role of the PKA regulatory function of SWS in OP-induced neuropathy and, based on our results propose a model in which OP-mediated changes in the PKA function of SWS lead to decreased PKA activity, which in turn induces behavioral deficits and degeneration. Interestingly however, this mechanism does not seem to play a role in acute toxicity of TOCP, as determined in primary neuronal cultures.

## Materials and Methods

### Fly stocks

All fly stocks were maintained and raised under standard conditions at 25°C. Canton S was used as wild type. UAS-SWS^R133A^ and UAS-PKA-C3 are described in [24], and *sws^1^* in [19]. *elav*-GAL4, UAS-dicer, and UAS-lacZ were obtained from the Bloomington stock center and *Appl*-GAL4 was kindly provided by L. Torroja (Universidad Autonoma de Madrid, Spain). The PKA-C3 RNAi line (Pka-C3^NIG.6117R^) was obtained from the National Institute of Genetics (NIG-Fly), Japan.

### TOCP treatment of flies and primary neurons

Flies were starved of food and water for 4 h and then transferred to empty plastic vials deprived of normal food sources and nutrition was provided in the form of 5% glucose solution with or without TOCP (Chem Service Inc. or TCI America) on a piece of tissue. Flies were kept on this food for 1 d and then transferred to standard food. To test food uptake, we added food color CC22 (AmeriColor Corp.). For feeding flies, TOCP and paraoxon were mixed with ethanol 1∶5 and then diluted to the final concentration in glucose solution, for the cell culture experiments they were mixed with ethanol 1∶500 and diluted to the final concentration in media. The corresponding amount of ethanol (vehicle) was added in the controls. Vials and culture dishes were kept in the dark during the treatment period due to the light sensitivity of TOCP.

### Survival studies

Flies were collected after eclosion and treated with different concentrations of TOCP as described above. Flies were transferred to a standard food vial after treatment and the vials exchanged every 4 d. Dead flies were counted at 2 d, 7 d and 14 d. Originally males and females were tested separately but because they did not show a difference in the sensitivity, the data were combined. Flies were kept in groups of 10–15 flies. At least 5 independent tests were performed.

### Cell culture studies

Cultures were prepared from 3rd instar wandering larvae as described in [25] and plated onto glass coverslips coated with 2 mg/ml Laminin and 0.2 mg/ml Concavalin A (both from Sigma). After plating, neurons were allowed to settle for 12 h before TOCP treatment and kept in the dark before taking pictures. For a blind analysis of neurite length, images were taken and the longest neurite of each cell measured using ImageJ and varicosities counted before revealing the genotype or treatment condition. The obtained pixel number was then converted into µm. Statistics were done using the GraphPad Prism program and one-way ANOVA with a Dunett's post test to compare several samples to a control and Student's t-tests when comparing two samples. A generalized linear model (GLM) was used to determine the interaction between genotype and treatment condition. Dendrite length was noted to be positively skewed and was model as following a gamma distribution (with log-link).

### Time-lapse imaging

Cells were prepared as described above but plated onto cell culture dishes and observed with an inverted microscope. Imaging began 30 mins after plating and a phase contrast picture was taken every 10 min for 16 h. Images were acquired using the MetaMorph software from Molecular Devices.

### Tissue sections and measurement of vacuolar pathology

Paraffin sections were performed as described in [24]. To quantify the vacuolization, we photographed the section that showed the worst phenotype without knowing the genotype or whether they were treated or not. The area of the vacuoles was then calculated in Photoshop as total pixel number, converted into µm^2^, and the genotype/treatment revealed. Statistical analysis was done using one-way ANOVA when multiple groups were compared and Student's t-tests in cases were only treated versus untreated flies of one genotype were compared. Numbers of analyzed flies are indicated above the bars in every figure (a minimum of 14 flies were analyzed). To determine the interaction between genotype and treatment condition when comparing control and *sws^1^* heterozygous flies we used flies that did show vacuolization. GLM was model following a gamma distribution (log-link).

### Esterase, AChE, and PKA activity assays

Esterase/phospholipase activity measurement was done as described in [24]. The AChE assay was done following the protocol described in [26] homogenizing 5 flies in 250 µl buffer and using 20 µl of this solution per experiment. PKA activity measurements in flies head extracts were done as described in [24]. Hippocampal neurons were provided by Dr. Banker and were prepared from E18 rats as described in [27] and treated 3 h after plating. For PKA assays, the media was removed and the cells scraped from the culture dish using 200 µl PKA extraction buffer (PepTag assay, Promega). All measurements were normalized to total protein levels in the sample as determined by Bradford assays. For the measurements with PKA-C3 and SWSR^133A^ expression, we normalized the values to untreated wild type that were included in each independent experiment due to variations between the different tests.

### Fast phototaxis and RING assays

Fast phototaxis assays were conducted in the dark using the countercurrent apparatus described by [28] and a single light source. A detailed description of the experimental conditions can be found in [29]. Flies were starved overnight, but had access to water and were tested the following morning. Five consecutive tests were performed in each experiment with a time allowance of 6 seconds to make a transition towards the light and into the next vial. The RING assays were performed as described in [30] with a time allowance of 6 s to climb up 7 cm into the next tube. In this assay, only a single try was analyzed. A minimum of eight independent tests with groups of 10–20 flies was used per genotype and treatment condition for each of the two assays. Statistical analysis was done using one-way ANOVA and Student's t-tests. GLM was used to determine the interaction between genotype and treatment condition when comparing control and SWS overexpressing flies with the response treated as following a normal distribution.

### Two-Hybrid assay

The Two-Hybrid-Screens were performed using the CytoTrap Vector kit from Stratagene (now Agilent Technologies) which is based on the activation of the Ras signaling pathway, thereby allowing cell growth. For the generation of the NTE bait construct, a cDNA construct encoding the full-length mouse NTE was obtained from a λgt11-library from BALB/c adult brain (Clontech) as we described in [9] and cloned into the pSOS vector. The target constructs were constructed by cloning cDNAs for the PKA-C1, PKA-C2, and PKA-C3 subunits (kindly provided by D. Kalderon, Columbia University, New York) into the pMyr vector as we described in [24]. pSOS-NTE was co-transformed into the cdc25H yeast strain with each of the pMyr-PKA-Cs or with the empty pMyr vector as a negative control using the protocol described in the kits user manual. Similarly, each of the PKA-Cs was co-transformed with the empty pSOS vector as negative controls (a positive control provided in the kit was also included). The cdc25H mutation prevents growth at 37°C whereas they are able to grow at 25°C. Only when the Ras pathway is activated by an interaction between the bait and target protein, cells can grow at the restrictive temperature. Each transformation was plated onto two glucose containing plates and two galactose containing plates and one each incubated at 37°C and 25°C. The two plates at the permissive temperature were used to determine the transformation efficiency of each combination whereas the glucose containing plate at 37°C was used to confirm that the mutant yeast cells had not reverted to the wild type. The galactose containing plate at 37°C was used to identify interactions because both, pSOS and pMyr contain a GAL1 promotor to induce the bait and target protein. Whereas the negative controls and the pSOS-NTE co-transformed with pMyr-PKA-C1 and pMyr-PKA-C2 only grew at 25°C, co-transformants with pSOS-NTE and pMyr-PKA-C3 grew at 37°C when galactose was provided but not with glucose confirming that the growth was not due to revertants. Single colonies from all co-transformants were picked and retested to confirm the interaction.

### Western blots

Heads from 2–3 d old flies were homogenized as described in [31] and proteins separated on 8% or 12% SDS-PAGE gels and transferred using the Bio-Rad Mini Trans-Blot® Cell system. Proteins were transferred to Hybond membranes (Amersham Bioscienses). The antibody against SWS [24] was used 1∶1000 and anti-PKA-C3 (kindly provided by B. Biteau, University of Rochester, New York) 1∶4000. All antibodies were diluted in TBST supplemented with 3% BSA. Bands were visualized using horseradish peroxidase-conjugated secondary antibodies (Jackson ImmunoResearch) at 1∶1000 and the SuperSignal® West Pico chemiluminiscent substrate (ThermoScientific). Homogenates from 10 fly heads were used per lane.

## Results

### Tri-ortho-cresyl phosphate inhibits SWS catalytic activity in flies

**Figure 1:**
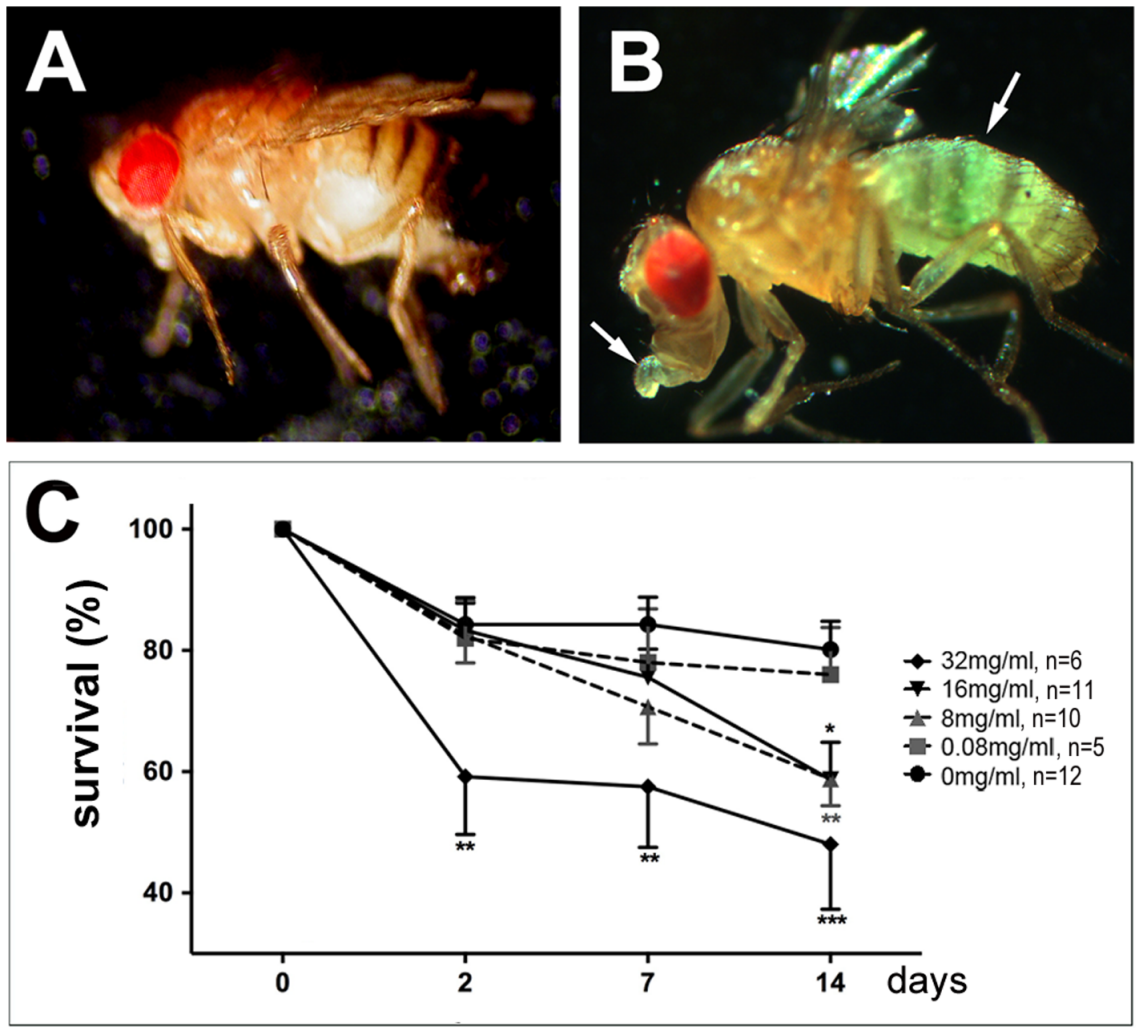
TOCP treatment induces lethality. **A**. Wild type fly fed on glucose. **B**. A wild type fly fed with glucose containing TOCP and blue food coloring shows food uptake by the blue coloring of the abdomen and proboscis (arrows). **C**. Survival of flies treated with different concentrations of TOCP. n =  number of independent tests with 10–15 flies. Analysis was done using one-way ANOVA with a Dunett's post test to compare the treated flies to mock treated flies. The SEMs are indicated. *p<0.05, **p<0.0. (the variance was not significantly different with the exception of day 14 with p = 0.02)

To establish *Drosophila* as a model for OP-induced neurotoxicity, flies were treated with the organophosphorous compound tri-ortho cresyl phosphate (TOCP). We chose TOCP because, in contrast to most other organophosphates, TOCP appears to have minimal or no effects on acetylcholine esterase (AChE) activity [18]. In addition, TOCP is the compound that originally lead to the identification of OPIDN (Smith, 1930) and it has been used to experimentally induce OPIDN in chickens [17]. We first determined an appropriate dose by treating flies with increasing doses of TOCP, ranging from 0.08 mg to 32 mg per ml glucose solution, which is approximately 0.5 to 150 times the neuropathic oral dose for humans with a fly weighing approximately 1 mg and drinking about 1 µl in the 1 d treatment period [32]. To confirm that the flies consumed the TOCP containing glucose solution, we added food color which was easily detectable after feeding (Fig. 1B, untreated fly in Fig. 1A), confirming that the flies did take up the food. Whereas the lowest concentrations of 0.08 mg/ml did not result in an increased lethality compared to mock treated flies (Fig. 1C), intermediated doses of 8 mg/ml and 16 mg/ml caused a significant increase in lethality after 14 d. These doses did not induce significant lethality after 2 d, showing that they had a normal survival rate during the treatment period. In contrast, the highest dose of 32 mg/ml resulted in the death of 40% of the flies already two days after treatment and 50% after two weeks (Fig. 1C). Although we cannot distinguish whether the lethality is solely due to neuropathologic effects or to effects of TOCP on other tissues, these results show that TOCP treatment is deleterious to flies.

**Figure 2:**
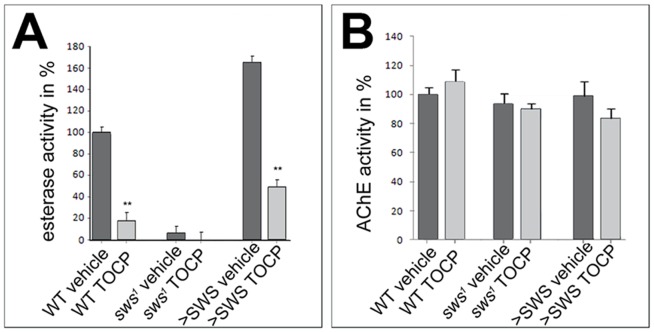
TOCP treatment inhibits SWS-esterase activity, but not AChE activity. **A**. Esterase activity against phenyl valerate is significantly inhibited by TOCP treatment (16 mg/ml) in wild type whereas the residual activity in *sws^1^* is not further reduced by TOCP. Flies overexpressing SWS using *elav*-GAL4 show approximately a 1.6 fold increase in activity in vehicle treated flies compared to wild type. Although this activity is significantly reduced by TOCP, the SWS overexpressing flies still show 50% of the activity of untreated wild type. Two independent measurements were done for treated flies and four for untreated flies. **B**. Neither TOCP treatment nor SWS levels have a significant effect on AChE activity. Two independent measurements were performed for each genotype and treatment. **A, B**. Flies were tested at the end of the 16 h treatment period. All values are shown relative to untreated wild type. A Student's t-test was used to compare each treated group to its corresponding untreated one. SEMs are indicated; **p<0.01. (The variances were not significantly different between treated and untreated flies for each genotype).

To induce OPIDN in chickens, NTE esterase activity must be at least 70% inhibited [11], [12]. We therefore measured to what degree TOCP inhibits SWS esterase activity at 16 mg/ml TOCP, a dose at which the majority of flies still survive and which therefore seemed appropriate for further experiments. As shown in figure 2A, the hydrolyzing activity against phenyl valerate, a standard substrate to detect NTE activity, was significantly reduced to about 20% (p<0.01) in head homogenates from treated wild type flies. To verify that this activity is indeed mediated by SWS, we included the *sws^1^* null mutant [19] which exhibited only a very low level of intrinsic esterase activity. As expected, this residual activity was not further inhibited by TOCP. Additional expression of SWS in neurons via the pan-neuronal *elav*-GAL4 driver line increased the esterase activity to approximately 160% of the wild type activity in untreated flies. Although TOCP treatment of the SWS overexpressing flies also significantly inhibited the esterase activity to 33% compared to the vehicle (p<0.01), the treated SWS overexpressing flies still retained 49% of the activity of untreated wild type flies, which is 2.5 times more activity as treated wild type. Measuring the AChE activity in these flies confirmed that TOCP treatment did not affect AChE activity nor did the levels of SWS (Fig. 2B) even at the highest dose of 32 mg/ml TOCP (Fig. S1).

### TOCP treatment induces neuronal degeneration and behavioral deficits

**Figure 3:**
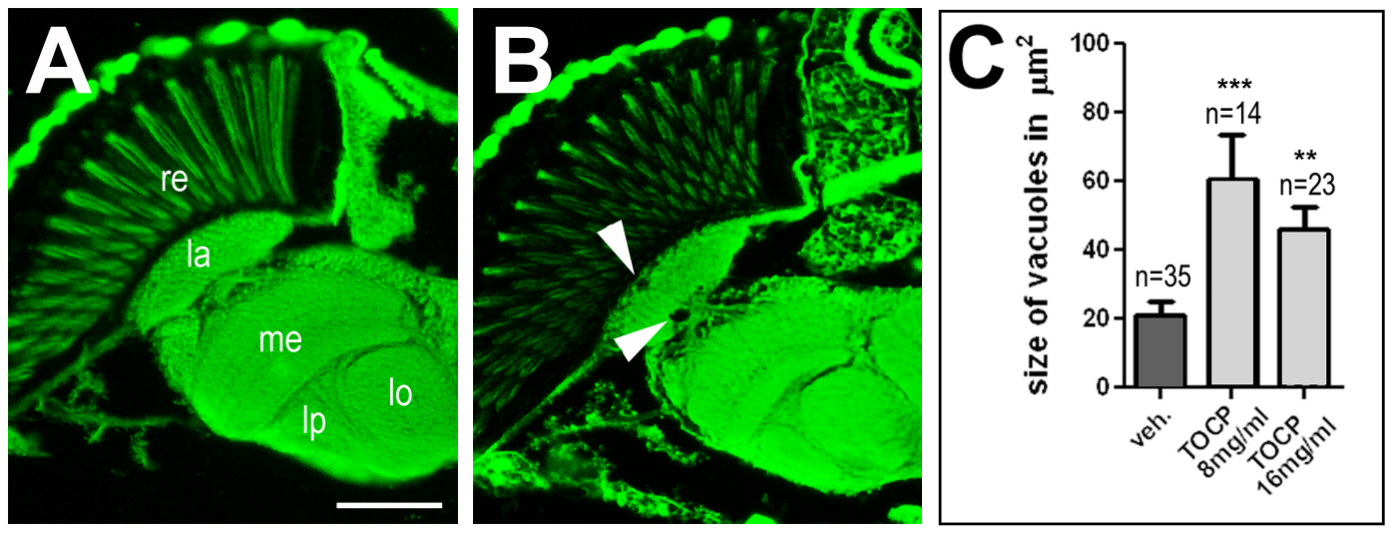
TOCP treated flies show neuronal degeneration. **A**. A paraffin head section of a wild type fly 14 d after vehicle treatment does not show overt degeneration. **B**. In contrast, a few vacuoles (arrowheads) have formed in an age-matched fly treated with 16 mg/ml TOCP. **C**. Measuring the area of vacuoles in the brain revealed a significant increase in 14 d old flies treated with 8 mg/ml or 16 mg/ml TOCP. Analysis was done using one-way ANOVA with a Dunett's post test to compare to vehicle treated flies. SEMs are indicated, n = number of flies analyzed; **p<0.01, ***p<0.001. Scale bar in **B** = 2 µm. re = retina, la = lamina, me = medulla, lo = lobila, lp = lobula plate. Scale bar in **A** = 40 µm. (The variance was not significantly different).

To determine whether TOCP treatment can induce degenerative phenotypes in flies, we treated 1 d old flies with the two intermediate doses (8 mg/ml or 16 mg/ml) of TOCP and examined them after 14 d. In contrast to mock treated flies (Fig. 3A), we could indeed detect spongiform lesions in TOCP treated flies (Fig. 3B, arrowheads). To quantify this phenotype, we measured the area of vacuoles which confirmed a significant increase with 60.5±13.2 µm^2^ (p<0.001) in the flies treated with 8 mg/ml and 46.3±6 µm^2^ (p<0.01) treated with 16 mg/ml with compared to 20.9±4.2 µm^2^ in mock treated flies (Fig. 3C). We also detected significant vacuole formation in the thoracic ganglia of TOCP treated flies (Fig. S2).

**Figure 4:**
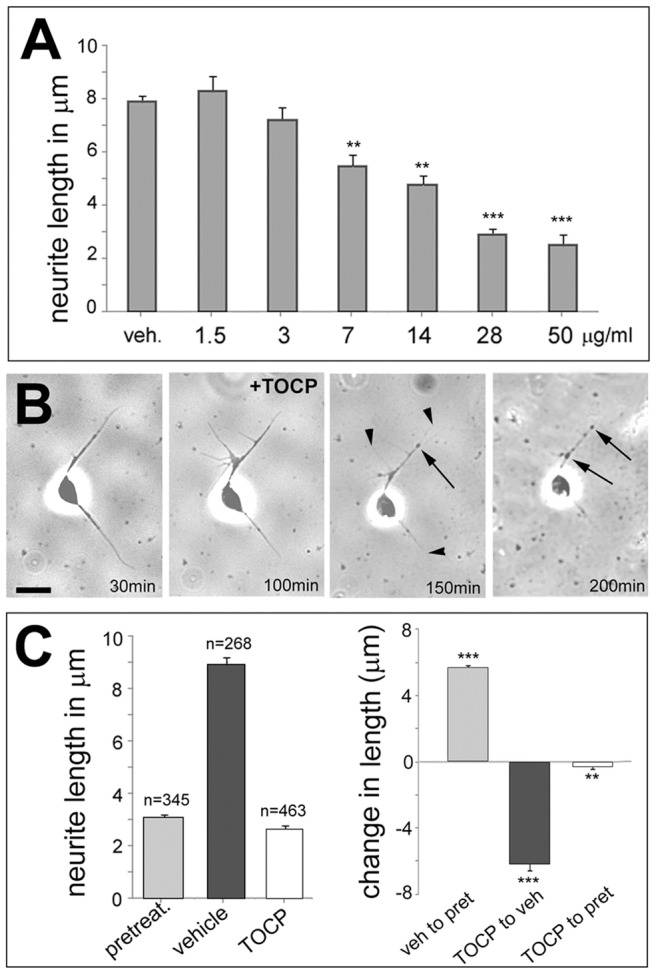
TOCP induces neurite shortening in primary neuronal cultures. **A**. Dose response curve showing that TOCP doses equal or higher than 7 µg/ml cause a significant reduction in neurite length. 30–56 neurons were measured for each condition. **B**. Live imaging of a neuron treated with 14 µg/ml TOCP reveals the formation of varicosities (arrows) and neurite degeneration (arrowheads) 50 min after the addition of TOCP. Another 50 mins later, these phenotypes are even more pronounced. **C**. The length of neurites is dramatically reduced in TOCP treated cells versus mock treated cells, but also significantly shorter in TOCP treated cells compared to cells that have been fixed at the time of treatment (left graph). The graph on the right shows the change in length between each condition. Analysis was done using one-way ANOVA with a Dunett's post test to compare to mock treated cells. n = number of cells measured and the SEMs are shown. **p<0.01, ***p<0.001. Scale bar in **B** = 2 µm. (The variances were significantly different between treated and untreated cells; p<0.001).

Although this revealed that TOCP treatment can lead to neuronal degeneration in flies, this experiment did not allow us to specifically detect axonal degeneration, which is a characteristic sign of OPIDN. We therefore performed primary neuronal cell cultures and treated them after 12 h in culture with different doses of TOCP for 6 h before measuring the longest neurite of each cell. Whereas the two lower doses did not affect the average neurite length when compared to vehicle treated cells, higher doses caused a significant reduction, which correlated with the increasing concentration of TOCP (Fig. 4A). To determine whether the addition of TOCP prevents further outgrowth or leads to retraction/degeneration of neurites, we performed live cell imaging studies. For this and the following studies, we chose the dose of 14 µg/ml which had an intermediate effect with approximately 40% reduction in neurite length in our dosage study. Also this dose did not induce a significant amount of cell death with 8.44±0.32% dead cells in treated neurons (n = 63) versus 7.9±2.7% in untreated ones (n = 58; p = 0.74). For live imaging, cells were allowed to adhere to a coverslip for 12 h, which was then transferred to the microscope chamber and allowed to settle for another 30 min before starting imaging. TOCP was added after 100 min and within 50 minutes of exposure, the neurites started to look thinner and shorter, followed by the formation of varicosities and fragmentation (arrows, Fig. 4B; the arrowheads point to shorter neurites), typical signs of Wallerian degeneration [33]. The percentage of cells with varicosities after TOCP treatment is shown in Fig. S3. In addition, this suggested that TOCP did not just prevent further outgrowth but caused the degeneration of existing neurites. To confirm this result, we fixed one set of neurons after 12 h in culture (pretreat. in Fig. 4C) whereas two other sets were treated with either 14 µg/ml TOCP or the vehicle (veh.). These neurons were then allowed to grow for another 6 h before the average neurite length was determined. As expected, neurite length was significantly shorter in the TOCP treated cells compared to vehicle treated ones, confirming the toxic effects of TOCP (Fig. 4C). But, whereas neurite length was dramatically increased in vehicle treated neurons compared to the ones fixed at treatment time (confirming their continuing growth), the average length of neurites from TOCP treated neurons was significantly shorter compared to the ones fixed 6 h earlier (Fig. 4C). This confirmed that TOCP treatment does result in a retraction/degeneration of established neurites in our model.

**Figure 5:**
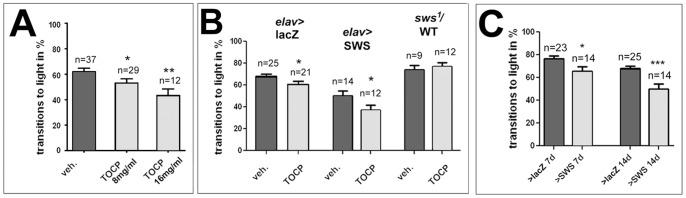
Effects of SWS levels on TOCP-induced behavioral deficits. **A**. Wild type flies treated with 8 mg/ml or 16 mg/ml TOCP show a significantly reduced performance in the fast phototaxis assay. **B**. A similar deficit is detectable in 14 d old control flies (expressing lacZ pan-neuronally; *elav*>lacZ) treated with 8 mg/ml TOCP. Expressing additional SWS pan-neuronally (*elav*>SWS) does not protect 14 d old flies from behavioral deficits caused by TOCP however heterozygosity for *sws^1^* (*sws*/WT) protects flies from the TOCP induced behavioral deficits (treated WT to *sws*/WT, *p<0.05). **C**. Comparing untreated flies in the phototaxis assay shows that overexpression of SWS alone results in less successful transitions already in 7 d old flies. This effect is even more severe when untreated 14 d old flies are tested. The analysis in **A** was done using one-way ANOVA with a Dunett's post test and the analyses in **B, C** with a Student's t-test, comparing treated and untreated flies of each genotype (in **B**) and control and SWS overexpressing flies of a given age (in C). n = is number of groups tested with 10–20 flies each. All flies were females. SEMs are indicated in all graphs. *p<0.05, ***p<0.001. (The was no significant difference in the variance in any of the comparisons).

In addition to axonal degeneration, OPIDN is characterized by locomotion deficits that lead to paralysis and spasticity, typically occurring a few weeks after exposure [4], [34]. To address this issue in flies, we performed fast phototaxis tests [28] of treated and untreated flies. Again 1 d old flies were kept on glucose solution with or without TOCP (8 mg/ml or 16 mg/ml) and then aged on normal food for 14 d. As shown in figure 5A, treatment of wild type flies with 16 mg/ml as well as with 8 mg/ml resulted in a significant decrease in performance when aged for 14 d with 53±3.2% transitions towards light for 8 mg/ml (p<0.05) and 43±5.1% for 16 mg/ml (p<0.01) compared to 63±3.2% in vehicle treated controls. For another behavioral test, we used RING assays which test how many flies walk upwards a vertical tube (7 cm) in 6 s. In this assay, we did however not get a significant difference between treated and non-treated flies (Fig. S4). This negative result could be due to the fact that the flies are less active in this assay, which does not induced a startle reaction, with only 35% transitions in contrast to 63% transitions to the next vial in the fast phototaxis assay within the same 6 s time frame. In addition, the flies are only performing a single transition in the RING assay whereas they are tested consecutively five times in the fast phototaxis assay and it is possible that the locomotion deteriorates over time and significant changes are therefore only detectable in the fast phototaxis assay. Alternatively, the deficits observed in the fast phototaxis assay are due to defects caused by effects on other cell types than motor neurons, including neurons in the brain and photoreceptors.

### Increased SWS levels do not protect against TOCP-induced phenotypes

As discussed above, OP treatment results in the inhibition of the esterase/phospholipase activity of NTE and this inhibition was proposed to be the cause of OPIDN. We therefore investigated whether increasing SWS activity can protect against TOCP toxicity. For this experiment, we induced SWS pan-neuronally via *elav*-GAL4 and to provide a comparable genetic background control for the GAL4/UAS [35] based overexpression studies, we used flies that contained the *elav*-GAL4 driver construct as well as an unrelated UAS construct (UAS-lacZ, expresses beta-galactosidase) as controls. Because the phototaxis assay is based on testing groups of flies and therefore requires large numbers of flies, we performed the following experiments only with one concentration; 8 mg/ml. Testing the control flies after 14 d of aging, the percentage of flies that managed to make the transition towards the light source was again significantly reduced to 61±2.7% in TOCP treated flies compared to the mock treated controls with 68±2.2% (p<0.05, Fig. 5B). Expressing additional SWS which, as shown in figure 2A, resulted in a 2.5 fold increase in the esterase activity after TOCP treatment compared to treated wild type flies, did not protect from TOCP-induced behavioral deficits in 14 d old flies. In fact it resulted in an even stronger reduction in performance (to 37±4.6% versus 50±4.3% in untreated flies; Fig. 5B), with a decrease of 26% between treated and untreated SWS overexpressing flies, whereas the difference was only 10% in control flies. Moreover, the additional expression of SWS caused a significant decrease in performance even in untreated flies, which was already detectable in 7 d old flies (66±3.4% versus 77±2.5%) and increased with further aging (Fig. 5C). Although we did see a slight increase change in the degenerative defects in untreated SWS overexpressing flies (see next paragraph), this was not statistically significant. However, analyzing sections from these flies by electron microscopy revealed abnormal mitochondria that appeared swollen and disorganized (Fig. S5) suggesting that there are neuronal defects in these flies. Although treated SWS overexpressing flies were significantly different from treated control flies, the change in performance of 26% to 10% (interaction) was not statistically significant. In contrast to the SWS overexpressing flies, heterozygous *sws^1^*/WT flies were protected from the deleterious effects of TOCP (74±4.1% in untreated versus 77±3.4% in treated 14 d old flies; Fig. 5B) although we previously showed that they have a 50% reduction in SWS activity [24]. Comparing the treated heterozygous *sws^1^* flies to treated controls (*elav*>lacZ) showed a highly significant difference with a p-value of 0.0008. In this case, untreated heterozygous *sws^1^* flies were not significantly different from untreated control flies, showing that reducing SWS esterase activity had no adverse effect on the behavior in the fast phototaxis assay.

**Figure 6:**
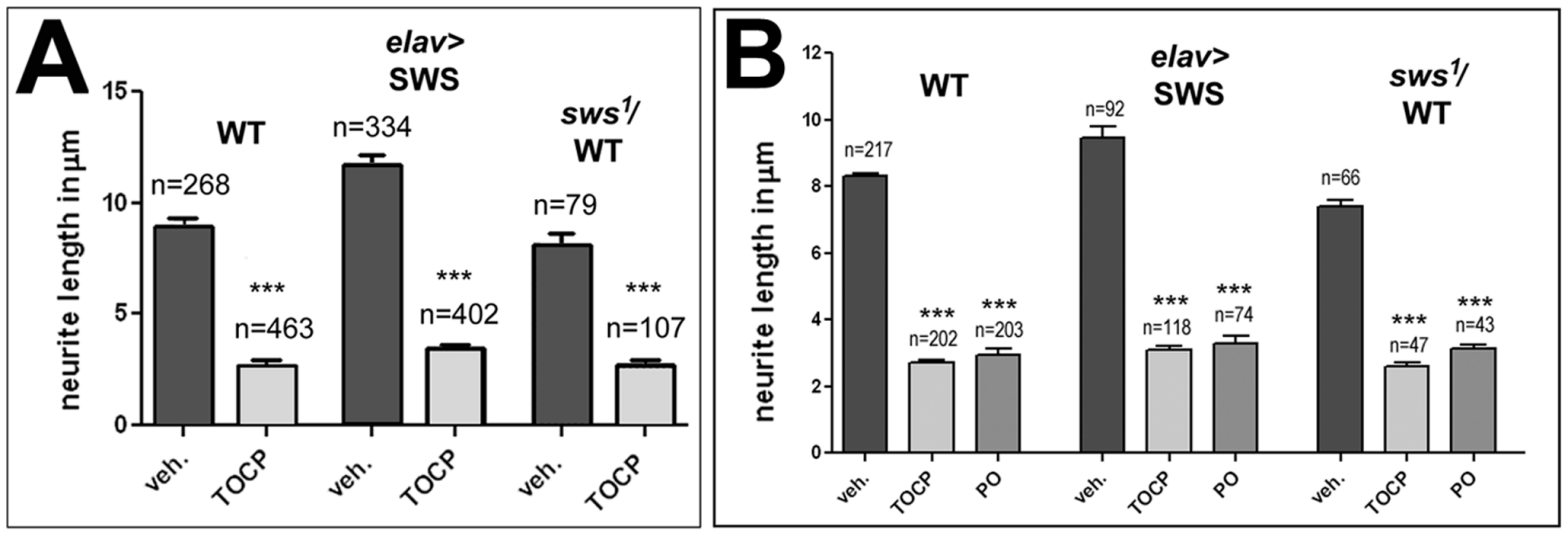
SWS levels do not affect axonal shortening. **A**. Neurite length was significantly shorter in all TOCP treated (14 µg/ml) cells and SWS levels had no significant effect on this TOCP-induced phenotype. **B**. Paraoxon (PO, 4 µg/ml) treatment resultin similar neurite shortening as TOCP treatment. A Student's t-test was used to compare treated and untreated cells of each genotype in **A**. One-way ANOVA and Dunett's post test was used for **B**. n = number of cells analyzed and SEMs are indicated. ***p<0.001. (The variances were significantly different between treated and untreated cells; p<0.001).

As a second assay to determine the effects of SWS levels on TOCP-induced phenotypes, we treated primary neurons derived from SWS overexpressing flies and flies heterozygous for *sws^1^* with TOCP at a dose of 14 µg/ml as described above. As shown in figure 6A, TOCP treatment significantly reduced neurite length in all genotypes (p<0.001), however, changing the levels of SWS had no significant effect on the sensitivity to TOCP. Surprisingly, expression of additional SWS significantly increased neurite length in untreated neurons (Fig. 6A), suggesting that SWS may play a role in neuronal outgrowth as well as in neuronal survival (even taking this difference into account, the interaction between the differences of various genotypes was not significant different). Because we did not observe any effects when altering SWS levels, we tested whether the neurite phenotype could be independent of SWS activity. We therefore treated these cells with the non-neuropathic paraoxon which is a potent AChE inhibitor that causes acute neurotoxicity but not the delayed toxicity characteristic for OPIDN. Indeed, treatment with 4 µg/ml did induce a comparable neurite shortening as TOCP treatment (Fig. 6B), suggesting that the acute effect of TOCP in these neurons could be independent of SWS. Unfortunately, we could not determine whether the effect is due to an inhibition of AChE or another target because these neuronal cultures did not provide sufficient material for AChE activity measurements. However our measurements in flies shown in figure 2A suggest that it might act on another target because AChE activity was not affected by TOCP when using fly extracts.

**Figure 7:**
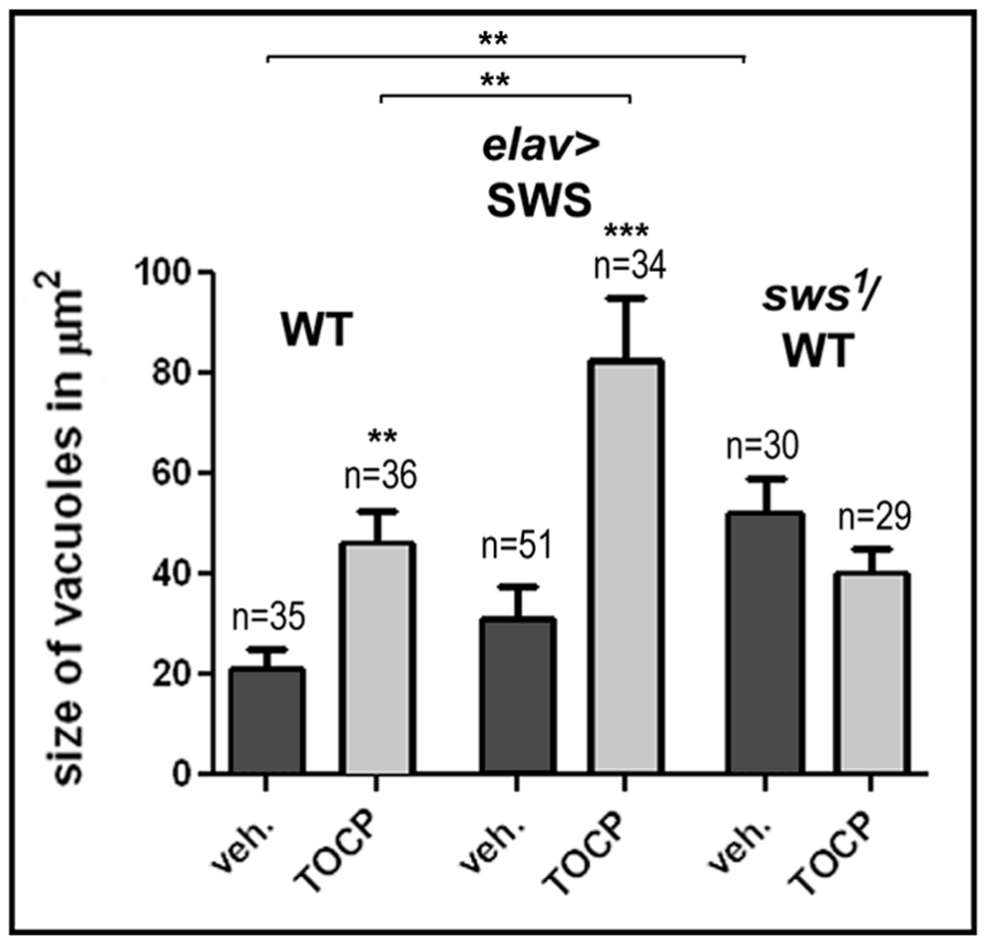
Effects of SWS levels on vacuole formation. Measuring the vacuole area in head sections from 14(16 mg/ml) wild type flies and SWS overexpressing flies. Untreated SWS overexpressing flies were not significantly different from untreated wild type but treated overexpressing flies revealed significantly more vacuolization. In contrast, heterozygous *sws^1^* flies, although showing significantly more vacuolization compared to wild type when untreated, where protected from TOCP-induced degeneration. A Student's t-test was used to compare treated and untreated flies of each genotype and to compare treated wild type and treated SWS overexpressing flies. n = number of sections analyzed and SEMs are indicated. **p<0.01, ***p<0.001. (The variances were significantly different when comparing treated SWS overexpressing flies with untreated overexpressing flies or with treated wild type; *p<0.05).

Lastly, we studied how SWS levels affect the degenerative phenotype caused by treating with 16 mg/ml TOCP. As in wild type, exposure to TOCP caused an increased vacuolization with 82.7±12.3 µm^2^ in treated SWS overexpressing flies compared to 31.1±6.5 µm^2^ in untreated ones (Fig. 7). This was also significantly more than in treated wild type with 46.3±6 µm^2^ (p<0.01), showing that additional expression of SWS significantly aggravates the degenerative phenotype. In contrast, heterozygousity for *sws^1^* did not alter the vacuolization after TOCP exposure when compared to treated wild type (39.9±5 µm^2^). However the mock treated *sws^1^*/WT flies had significantly more vacuolization with an area of 52±7.1 µm^2^ than mock treated wild type flies (p<0.01; Fig. 7). This reveals that heterozygous *sws^1^* flies have a weak degenerative phenotype at this age, which is however much weaker than in age-matched homozygous *sws^1^* mutants, which are riddled with vacuoles (3784±804.8 µm^2^). Taking this increase in vacuolization in untreated flies into account, heterozygosity for *sws^1^* actually protects against effects of TOCP (Fig. S6), correlating with the protective effect in the behavioral tests. Together these experiments show that increased levels of SWS, and the resulting increase in esterase/phospholipase activity, does not protect against TOCP-induced toxicity, but instead aggravated the delayed phenotypes but not the acute toxicity of TOCP in cell culture. In contrast, reducing SWS, though reducing the esterase/lipase activity, had a protective effect on the delayed symptoms. This suggested that heterozygous flies may be protected because they make less toxic, TOCP-modified SWS, supporting previous observations that a gain-of function mechanism of aged SWS/NTE plays a role in the development of TOCP-induced phenotypes.

### TOCP treatment interferes with PKA activity

**Figure 8:**
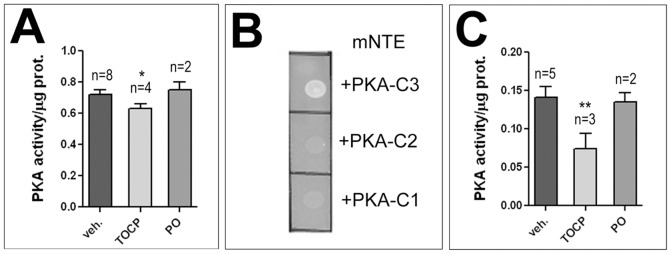
TOCP treatment reduces PKA activity. **A**. Flies treated with 16 mg/ml TOCP reveal a significant reduction in PKA activity in head extracts whereas flies treated with Paraoxon (PO; 0.2 mg/ml) show no change in PKA activity. **B**. Mouse NTE binds fly PKA-C3 in Two-Hybrid assays, whereas it does not interact with the other two known fly catalytic subunits, PKA-C2 and PKA-C1. **C**. TOCP treatment (14 µg/ml) reduces PKA activity in cultured rat hippocampal neurons whereas exposure to Paraoxon (PO; 4 µg/ml) does not. n = is number of independent PKA assays. PKA activity is given as the ratio of the luminosity value of phosphorylated to unphosphorylated kemptide peptide per µg protein. Student's t-tests were used to compare treated and untreated flies or hippocampal neurons. SEMs are indicated. *p<0.05, **p<0.01. (The variances are not significantly different).

We previously showed that SWS, besides acting as a phospholipase, also binds the C3 catalytic subunit of PKA, thereby inhibiting PKA activity [24]. We therefore tested whether the PKA function is affected by TOCP treatment. Measuring PKA activity in head extracts from flies fed with 16 mg/ml TOCP for 24 h resulted in a significant decrease in PKA activity when compared to vehicle treated flies (Fig. 8A), suggesting that TOCP interferes with the release and activation of PKA-C3. In contrast, treating flies with the non-neuropathic paraoxon (0.2 mg/ml, the flies did not survive higher doses) did not result in decreased PKA activity in two independent measurements (Fig. 8A). To determine whether this effect could also play a role in the human syndrome and because the PKA function had not been confirmed for vertebrate NTE, we performed Two-Hybrid assays with mouse NTE (mNTE). Using the three catalytic subunits known in *Drosophila* showed that mNTE binds PKA-C3, but not the other two catalytic subunits (Fig. 8B). This shows that vertebrate NTE, like SWS [24], specifically binds to the C3 subunit, further confirming the functional conservation of SWS and NTE. After verifying that NTE can interact with the PKA-C3 catalytic subunit, we treated cultured hippocampal neurons derived from rats with TOCP and measured the effect on PKA activity (rat NTE is 99% identical to mouse NTE and 95% identical to human NTE). Treating these neurons with a dose of 14 µg/ml TOCP for 1 d also resulted in a significant reduction in PKA activity (Fig. 8C) suggesting that TOCP-induced changes in the activity of the vertebrate orthologues of PKA-C3, Pkare in mouse and PrKX in humans, is part of the toxic mechanism of TOCP (the mouse and the rat protein are 95% identical). Again, treatment with paraoxon at 4 µg/ml had no effect on PKA activity. The effect of TOCP exposure on PKA activity was not due to an increased lethality of cells because treatment resulted in 9.44±0.32% dead cells versus 7.9±2.7% in mock treated cells (p = 0.74). Neither was this effect due to an effect on PKA-C3 levels because treatment with TOCP did not change the levels of PKA-C3 nor SWS (Fig. S7).

### Increased levels of PKA-C3 are protective against TOCP-induced deficits

**Figure 9:**
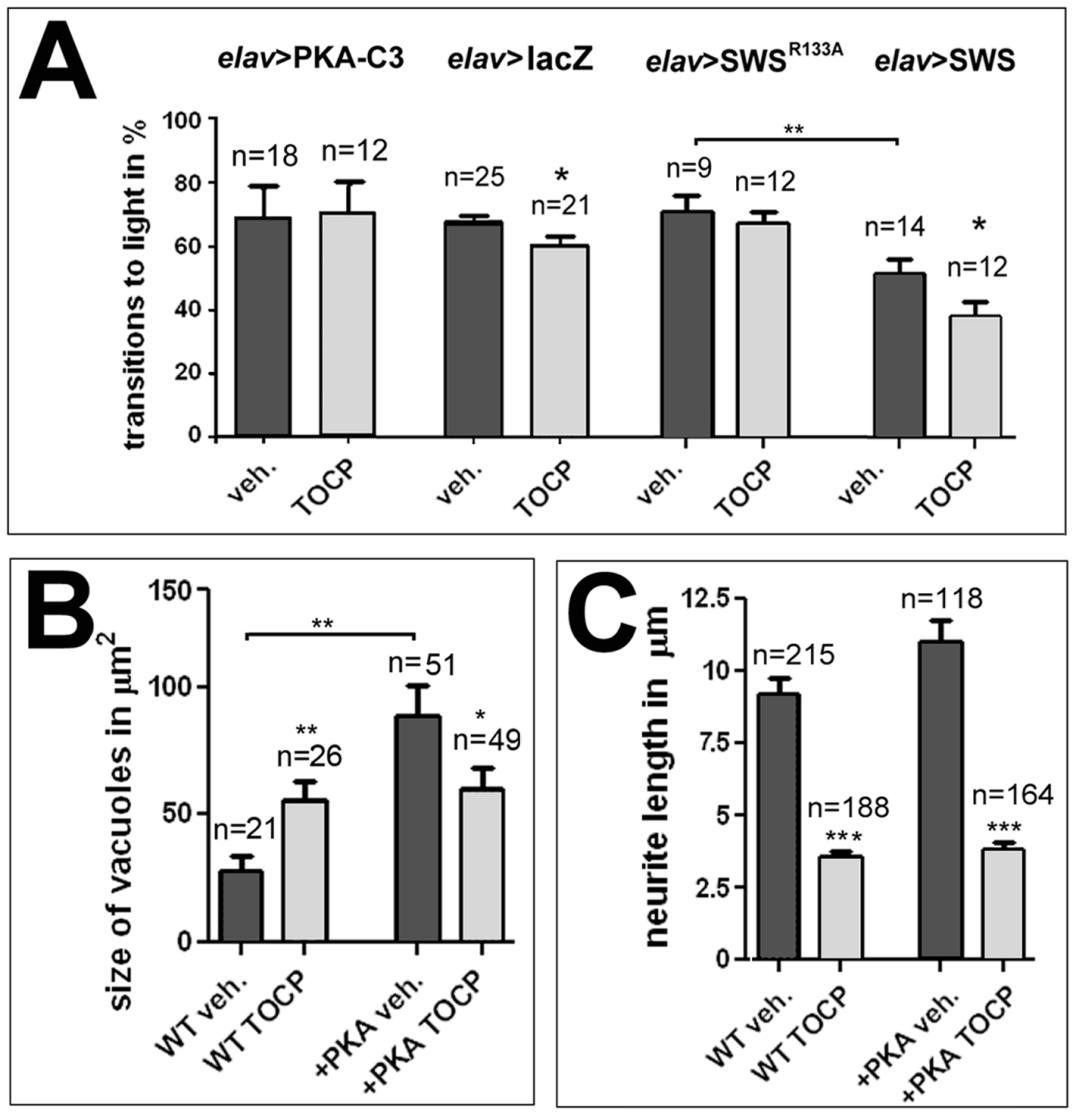
PKA-C3 overexpression protects against TOCP-induced degeneration and behavioral deficits. **A**. Flies expressing additional PKA-C3 in neurons via *elav*-GAL4 do not show the TOCP-induced reduction in performance seen in *elav*>lacZ control flies. Also flies expressing the PKA-C3 binding deficient SWS^R133A^ construct are protected against TOCP-induced behavioral deficits. In addition, these flies do not show the reduction in performance observed in untreated flies overexpressing the wild type SWS construct (*elav*>SWS). **B**. Although PKA-C3 overexpressing flies show a significant increase in vacuole formation when untreated, TOCP treatment does not enhance this phenotype, but significantly reduces vacuole formation. **C**. PKA-C3 overexpression has no effect on the neurite shortening observed after TOCP treatment of primary neurons. n = is number of groups tested with 10–20 female flies each in **A**, n = number of cells or head sections analyzed in **B** and **C**. Student's t-tests were used to compare treated and untreated flies and to compare untreated SWS and SWSR^133A^ overexpressing flies. A student's t-test was also used to compare vacuole size in untreated PKA-C3 overexpresing flies with controls in **B**. All flies used in the fast phototaxis assays were 14 d old females. SEMs are indicated in all graphs. *p<0.05, **p<0.01, ***p<0.001. (The variances were not significantly different in the tests done to compare vacuole size and behavioral deficits, but were different between treated and untreated cells: p<0.001).

The results described above suggest that a decrease in PKA-C3 activity plays a role in the behavioral and degenerative defects occurring after TOCP exposure. To further support this, we treated flies that expressed additional PKA-C3 in neurons via *elav*-GAL4 with 8 mg/ml TOCP as described above and performed fast phototaxis assays. As shown in figure 9A, these flies were protected from behavioral deficits with a performance index of 71±4.1%, which is not different from the value of untreated PKA-C3 overexpressing flies (69±3.0%) or untreated control flies (*elav*>lacZ; 68±2.2%).

As we showed previously, additional pan-neuronal expression of PKA-C3 not only enhanced the degenerative phenotype of *sws^1^*, but also caused vacuole formation on its own [24] and measuring the vacuole area in 14 d old mock-treated PKA-C3 overexpressing flies confirmed this result (Fig. 9B). Treating these flies with TOCP showed that the additional PKA-C3 was not only protective, but actually significantly reduced vacuole formation from 86.3±13.4 µm^2^ to 58.1±8.3 µm^2^. This suggests that reducing PKA-C3 activity by TOCP treatment can counterbalance the negative effects caused by overexpression of PKA-C3. Finally, we tested whether additional expression of PKA-C3 can ameliorate the effect of TOCP on neurite length in cell culture. However, as shown in figure 9C there was no significant difference in TOCP-induced neurite degeneration when comparing wild type and PKA-C3 overexpressing neurons.

To support our hypothesis that the effect of TOCP on PKA-C3 is mediated by SWS, we used a mutated form of SWS. In this construct (SWS^R133A^), an arginine that is required for the binding to PKA-C3 is replaced by alanine substantially reducing PKA-C3 binding efficiency [24]. In contrast to the pan-neuronal expression of wild type SWS protein, which significantly reduced performance in untreated flies (50±4.3% compared to untreated *elav*>lacZ controls with 68±2.2%), expression of this construct had no adverse effect on the performance in the phototaxis assay with 69.1±4.8% successful transitions (Fig. 9A). In addition, *elav*>SWS^R133A^ flies were protected from the toxic effects of TOCP treatment (65.3±3.9%) whereas flies overexpressing the wild type form were not (Fig. 9A). That the PKA-C3 binding-deficient SWS does not show behavioral deficits after TOCP treatment further supports our model that increased binding of SWS to PKA-C3 and the resulting decrease in PKA-C3 activity is playing an important role in TOCP toxicity.

**Figure 10:**
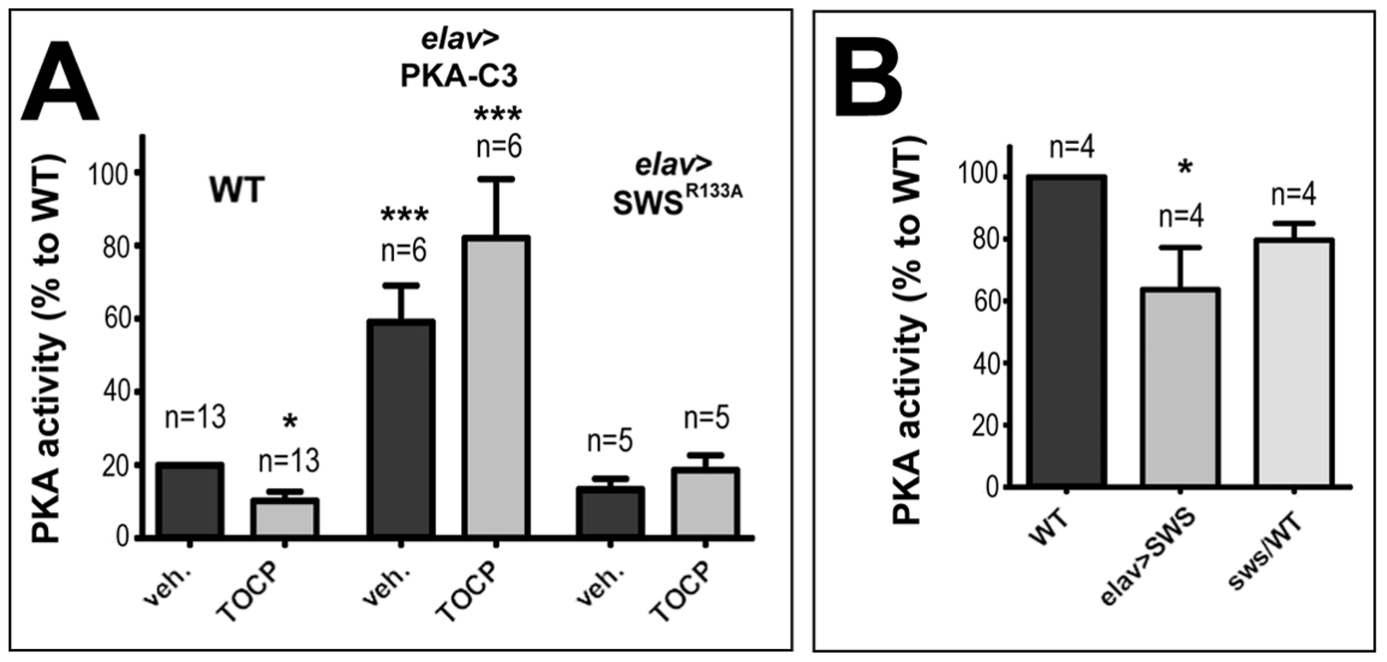
PKA-C3 and SWSR^133A^ expression prevent TOCP-induced reduction in PKA activity. **A**. PKA-C3 overexpression increases PKA activity and this is not affected by TOCP treatment. **B**. Additional expression of SWS significantly reduces PKA activity whereas heterozygosity for *sws^1^* does not. The values are shown in percent of the PKA activity in untreated wild type. n = number of independent measurement and the SEMs are indicated. Student's t-tests were used to compare activity in untreated and treated flies. One-way ANOVA with a Dunett's post test was used to compare the untreated flies in B. *p<0.05, ***p<0.001. (.The variances were not significantly different).

To determine how overexpression of PKA-C3 affected PKA activity, we performed measurements in treated and untreated flies expressing PKA-C3 via *elav*-GAL4. As shown in figure 10A, PKA-C3 expressing flies did indeed show a dramatic increase in PKA activity and this was not affected by TOCP treatment (the values were normalized to untreated wild type). In contrast, we did not see a change in the activity in untreated flies expressing SWS^R133A^ nevertheless they were protected from the TOCP induced reduction in PKA activity, in agreement with the protection observed in the behavioral assays. Finally, we measured PKA activity in SWS overexpressing flies and in *sws^1^* heterozygous flies and compared it to wild type. Although we did not detect a difference in the latter, the SWS overexpressing flies did show a significant reduction in PKA activity (Fig. 10B) which supports the hypothesis that expression of this construct induces behavioral deficits due to the increased binding and inhibition of PKA-C3.

### Knocking down PKA-C3 causes degeneration and behavioral deficits

**Figure 11:**
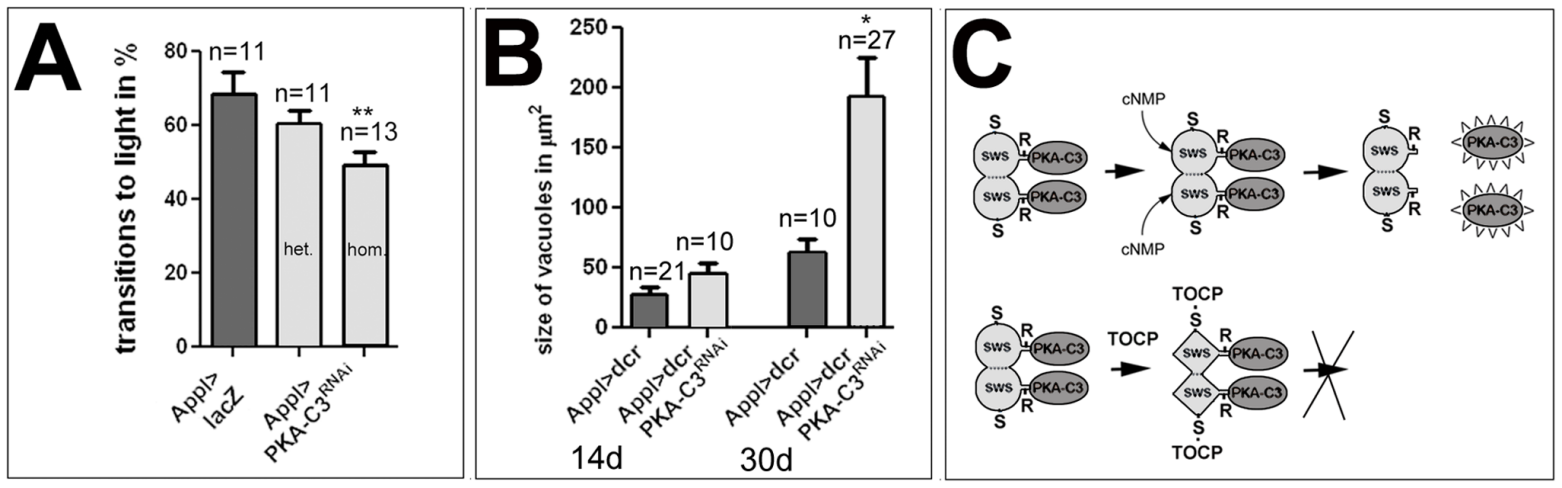
Loss of PKA-C3 causes behavioral and degenerative phenotypes. **A**. Inducing a UAS-PKA-C3^RNAi^ construct pan-neuronally with *Appl*-GAL4 (*Appl*>PKA-C3^RNAi^) did not result in a significant reduction in performance compared to controls (*Appl*>lacZ) when only one copy of each construct is used (het.). However, when using two copies of the RNAi construct (hom.), the flies performed significantly worse than controls. All flies were 14 d old females. n = is number of groups (10–15 flies) tested. Analysis was done using one-way ANOVA with a Dunett's post test and the variance was not different. **p<0.01. **B**. Measuring the vacuole area in 14 d old *Appl*>dcr;PKA-C3^RNAi^ flies did not reveal a significant difference to age-matched *Appl*>dcr controls. However, when aging the PKA-C3 knock-down flies for 30 d they showed significantly increased vacuolization compared to controls. n = number of analyzed flies. Student's t-test were used to compare vacuole size between flies of a given age. The variance was not significantly different when comparing 14 d old flies but were different when comparing 30 d old flies; p<0.001. *p<0.05. **C**. Model showing the interactions between SWS (light grey) and PKA-C3 (dark grey) and the proposed effects of TOCP. SWS binds to the PKA-C3 subunit via its interaction domain which contains the conserved arginine (R) that is required for binding and that is mutated in SWS^R133A^. S indicates the serine to which organophosphates bind. Canonical regulatory PKA subunits form dimers and release the catalytic subunits upon binding of cAMP. Although this has not been confirmed for SWS yet, potential cyclic nucleotide binding sites have been identified in SWS. In our model, binding of TOCP (or its metabolite SCOP) and the following aging reaction causes a conformational change that prevents the release and activation of PKA-C3, possibly by interfering with cyclic nucleotide binding.

If the decrease in PKA-C3 activity is causing TOCP-induced phenotypes, reducing PKA-C3 levels by genetic means should have a similar effect. To test this, we used an RNAi construct against PKA-C3 and expressed it pan-neuronally using *Appl*-GAL4. Performing fast phototaxis assays in 14 d old flies carrying on copy of the driver and one copy of the RNAi construct (*Appl*>PKA-C3^RNAi^ het.) did not reveal a significant difference to the control flies expressing lacZ (Fig. 11A). However, when we used flies with two copies of the RNAi construct (hom.), they performed significantly worse (49±3.4% versus controls with 69±5.8%). Performing qPCRs, we found a 22% decrease in PKA-C3 mRNA levels but due to the large variation and the resulting SEM of 31% this was not significant. Heterozygous knockdown flies showed a decrease of 38±12% and this was statistically significant to the controls (Fig. S8). Finally, we investigated whether PKA-C3 knock-down flies show vacuole formation. To increase the effectiveness of the knock-down, we also expressed dicer in these flies. Although 14 d old *Appl*-GAL4;UAS-PKA-C3^RNAi^;UAS-dcr flies showed more vacuolization the difference was not significantly different from *Appl*-GAL4;UAS-dcr control flies (44.7±8.7 µm^2^ versus 27.9±6.3 µm^2^). However, when aged for 30 d the knock-down showed more degeneration with a vacuole area of 192.1±32.5 µm^2^ compared to 63±11.4 µm^2^ in the controls (Fig. 11B; p = 0.023).

## Discussion

Organophosphate induced delayed neuropathy (OPIDN) was first described in the 1930s, then known as Jake leg syndrome because it was observed after the consumption of a beverage called Jamaica Ginger or Jake [4]. It was estimated that about 50,000 people were crippled with partial paralysis, due to Jake containing the organophosphorous compound tri-ortho-cresyl phosphate (TOCP) [4]. Other epidemics of TOCP poisoning and OPIDN have occurred in Morocco and India due to contaminated cooking oil [36], [37]. It was later found that TOCP acts on NTE/SWS and inhibits its function as an esterase/phospholipase [10]. However, several observations suggested that additional modifications of NTE/SWS might play a role in developing OPIDN. To address this issue, we used *Drosophila* to study the effects of TOCP on SWS, the fly orthologue of NTE.

TOCP can be metabolized into saligenin cyclic o-tolyl phosphate (SCOP), which has been proposed to be the active compound that actually binds and inhibits NTE [38]. We therefore first determined whether the toxic function of TOCP is conserved in flies. Feeding TOCP to flies indeed resulted in a reduced esterase activity, as has been described in vertebrates [7], [11]–[13]. As shown in figure 2A, we could achieve over 80% reduction in activity, a level sufficient to induce OPIDN in other models. Consistent with the role of SWS as the molecular target of TOCP, flies lacking SWS (*sws^1^*), which already show very low esterase activity, were not further affected by TOCP. On the contrary, flies expressing additional SWS showed increased esterase activity, which was significantly inhibited by TOCP, although they still showed significantly more activity (2.5 fold) than treated wild type. Examining brains and thoracic ganglia of TOCP treated flies confirmed the neurotoxic effects of this compound, which was further supported by studies in which primary neurons showed the signs of Wallerian degeneration that were also described in other models of OPIDN [33]. Finally, TOCP exposure caused behavioral deficits in flies which, as in vertebrates, is a delayed effect because we could not detect changes 7 d after treatment, whereas flies performed significantly worse 14 d after treatment. Together these experiments show that TOCP is neurotoxic in flies and has very similar effects as it has in vertebrates.

As described above, it has been shown that TOCP treatment inhibits the esterase/phospholipase activity of NTE and we have shown here that the same is true for SWS. To determine whether the reduction in phospholipase activity is sufficient to induce the toxic effects, we used flies with reduced and increased SWS levels. Surprisingly, increasing the amounts of SWS did not protect against TOCP-induced degeneration or behavioral deficits, but even increased the effects although performing a statistical analysis for the interaction of the genotype and the treatment did not reveal statistical significance. It has been shown in other species that NTE has to be inhibited by at least 70% [7], [11]–[13] to cause OPIDN and SWS overexpression flies still have 50% of the phospholipase/esterase activity found in wild type after TOCP treatment. If OPIDN results solely from reduced phospholipase activity, SWS overexpression and the resulting increase in activity should prevent or at least reduce the toxic effects after TOCP exposure. Furthermore, reducing the levels of SWS by treating heterozygous *sws^1^* flies did not increase the toxicity, but protected the flies form behavioral deficits and neurodegeneration.

That *sws^1^* heterozygous flies were protected from the delayed symptoms caused by TOCP and SWS overexpressing flies were more sensitive, at least when analyzing the degeneration, strongly suggested that the delayed phenotypes are caused by another function of SWS, than the phospholipase function. It also supported the hypothesis that these defects are caused by inducing a toxic gain-of function which is prevented when the amount of the toxic, TOCP-modified SWS is reduced, as in the case of *sws^1^* heterozygous flies. Surprisingly, changes in SWS levels had no effect on the TOCP-induced neurite shortening in primary neuronal cultures, suggesting that this acute phenotype is due to a different toxic mechanism of TOCP. Indeed, treatment with paraoxon, a potent AChE inhibitor that does not induce OPIDN resulted in a very similar neurite retraction, further supporting our assumption that mechanisms of the acute toxicity are different or might even not be mediated by NTE.

We previously described that SWS can act as a non-canonical regulatory subunit of protein kinase A (PKA) by binding and inhibiting the C3 catalytic subunit [24]. We therefore tested whether the gain-of function effect of TOCP on SWS could be due to altering the interaction of SWS with PKA-C3. Indeed, exposing flies to TOCP resulted in a significant decrease in PKA activity, a result that was confirmed for vertebrate NTE using rat primary neurons. Like SWS, rodent NTE did bind to the C3 subunit whereas it did not interact with the other two catalytic subunits of flies, suggesting that the PKA-regulatory function is conserved in vertebrate proteins. In this context, it is noteworthy to mention that vertebrates have a C3 orthologous catalytic subunit, called Pkare in mouse and PrKX in humans, and that these subunits are more conserved between the different species than they are related to different subtypes from one species [39]. As observed in flies, TOCP treatment of hippocampal neurons reduced PKA activity, suggesting that TOCP also interferes with the release and activation of the vertebrate catalytic subunits from NTE.

To obtain further support for our model that reduced PKA activity plays a role in the delayed symptoms of OPIDN, we used flies that expressed additional PKA-C3 and indeed this protected them from TOCP-induced behavioral and degenerative defects. Consistent with this result, PKA-C3 expression increased PKA activity and this was not significantly reduced by TOCP treatment. In addition, expressing a SWS construct with a mutation in the PKA-C3 binding site (SWS^R133A^), which we previously showed has a dramatically reduced binding capacity for PKA-C3 [24], also protected from these TOCP-induced phenotypes. This suggests that TOCP (SCOP) binding to the active site serine in the esterase domain and the following aging reaction not only inhibits the phospholipase function, but also modifies SWS in a way that hinders the release of PKA-C3, thereby inhibiting PKA activity (Fig. 11C). In the case of SWS^R133A^ overexpression, there would be no effect on PKA activity because this mutant SWS protein cannot bind and inhibit PKA-C3. In agreement with this model, flies expressing SWS^R133A^ did not show a decrease in PKA activity after TOCP exposure. Interestingly, expression of this construct also appeared to reduce the effects of TOCP on the endogenous wild type SWS. This might be due to sequestering the toxin because SWS^R133A^ has an intact phospholipase domain including the serine to which TOCP (SCOP) is bound permanently after the aging reaction occurred. Alternatively, SWS^R133A^ forms dimers with the endogenous SWS that cannot interact with PKA-C3, thereby reducing the availability of binding competent dimers that, when modified by TOCP do inhibit PKA-C3 (though dimer formation, as described for canonical PKA regulatory subunits, has not been confirmed for SWS). Our model is also in agreement with the observation that additional expression of wild type SWS already had an adverse effect on the performance in the fast phototaxis assay in untreated flies (which was not observed after overexpression of SWS^R133A^). In this case, the phenotype would be caused by increased PKA-C3 inhibition due to the excess of binding-competent SWS (we previously showed that increased SWS expression reduced PKA activity though the effect was not quite statistically significant with a value of p = 0.054; [24]). TOCP treatment can then further decrease PKA activity because the additional SWS can bind more PKA-C3 and is in addition not able to release it.

Lastly, we confirmed that decreasing PKA-C3 activity has similar effects as TOCP treatment because a pan-neuronal knockdown of PKA-C3 also resulted in behavioral deficits and vacuole formation. Based on these results we propose a model in which TOCP affects both, the phospholipase and PKA function of SWS/NTE. TOCP binding to the active site serine inhibits the phospholipase activity of SWS and through the following aging reaction, a side group is released from the bound OP and transferred within SWS, resulting in a conformational change of SWS that prevents the release of the PKA-C3 catalytic subunit and consequently its activation. In canonical PKA complexes the release of the catalytic subunit is due to the binding of cAMP to the regulatory subunit. Although regions with similarity to cyclic nucleotide binding sites have been found in SWS and NTE [19], [40], we currently do not know whether cyclic nucleotide binding regulates the release of PKA-C3 from SWS. Further studies are therefore needed to determine whether TOCP interferes with cyclic nucleotide binding or another aspect of release. Due to the known function of PKAs in transcriptional regulation, these delayed effects may be mediated by changes in the expression of downstream targets of PKA-C3 and the subsequent effect on the nervous system. However, currently not transcriptional targets of PKA-C3 have been identified and therefore future studies are needed to address this issue.

Because PKA-C3 overexpression did not prevent the acute effect of TOCP treatment on axonal degeneration in the cell culture experiments, this aspect of TOCP toxicity might be mainly caused by the loss of the phospholipase activity of SWS. That changing the levels of SWS had no effect on axonal shortening might be due to insufficient changes in phospholipase activity by our genetic manipulations, although it did increase or reduce esterase activity by 50%. Alternatively, the observed changes in axonal length are due to both, axonal shortening due to lack of the phospholipase activity, and the beginning of cell death due to the production of toxic modified SWS. In this case, additional SWS could ameliorate the first, but enhance the latter thereby not having an overall effect. However, the acute effect could also be due to TOCP acting on another target, an aspect that has to be explored in the future.

## Supporting Information

Figure S1 **AChE activity after treatment with 32 mg/ml TOCP.** Neither TOCP treatment nor SWS levels have a significant effect on AChE activity. Two independent measurements were performed for each genotype and treatment. Student's t-test was used to compare each treated group to its corresponding untreated one. SEMs are indicated. (The variances were not significantly different between treated and untreated flies for each genotype).
(TIF)

Figure S2 **TOCP treatment results in vacuole formation in the thoracic ganglia.** A. A paraffin section of a thoracic ganglia of a 14 d old wild type fly does not show vacuole formation whereas some vacuoles can be detected in a TOCP (32 mg/ml) treated fly (B). C. Measuring the area of vacuoles in the thoracic ganglia revealed a significant increase in TOCP treated flies. SEMs are indicated, n = number of thoracic ganglia analyzed; **p<0.01. Scale bar in in A = 15 µm. (There was no difference in variance).
(TIF)

Figure S3 **Varicosities are forming in cells treated with TOCP.** Counting the percentage of cells that have developed varicosities reveals that substantially more cells show this sign of Wallerian degeneration when treated with TOCP (14 µg/ml). n =  number of cells analyzed.
(TIF)

Figure S4 **TOCP treatment does not induce defects in RING assays.** Comparing untreated and TOCP treated flies in a RING assay did not reveal a difference in performance. Analysis was done with a Student's t-test and the SEM is indicated. n = is number of groups tested with 10–20 flies each. (There was no significant difference in the variance).
(TIF)

Figure S5 **Altered mitochondria morphology in SWS overexpressing flies.** A. A mitochondria in the axons shows abnormal cisternae and appears swollen (arrowhead) in comparison to normal looking mitochondria (arrows). B. Similar abnormal looking mitochondria can be found in neuronal cell bodies (arrowhead) whereas other mitochondria in the vicinity appear normal (arrows). Scale bar in A = 0.4 µm, in B = 0.8 µm.
(TIF)

Figure S6 **Interaction test to determine the effect of the genotype on the sensitivity to TOCP.** Due to the increased vacuolization a generalized linear model following a gamma distribution (log-link) was used to compare the changes in the difference between treated and untreated wild type flies (a) and treated and untreated *sws^1^* heterozygote flies (b). Only flies that showed vacuoles were used and the difference in the area of vacuoles was compared. This model revealed a significant difference with p = 0.018. n = number of sections analyzed.
(TIF)

Figure S7 **PKA-C3 and SWS protein levels are not affected by TOCP.** Neither treatment with 8 mg/ml or 16 mg/ml did reduce the amount of SWS or PKA-C3. A loading control using anti-Tubulin is shown below.
(TIF)

Figure S8 **Fold change in PKA-C3 mRNA in the knockdown.** Performing quantitative PCR we found a decrease in PKA-C3 mRNA levels in the heterozygous knockdown however this did not reach statistical significance. In contrast, the mRNA levels are significantly reduced in the homozygous knockdown. Triplicates of each genotype were used in each qPCR. Primers for actin were used as controls. SEMs are indicated, n = number of independent PCR reactions; *p<0.05.
(TIF)
