# Supplementary figures and images for: Organophosphate-Induced Changes in the PKA Regulatory Function of Swiss Cheese/NTE Lead to Behavioral Deficits and Neurodegeneration
Source: PLoS One. 2014 Feb 18;9(2):e87526. doi: 10.1371/journal.pone.0087526 (PMC3928115; doi:10.1371/journal.pone.0087526)

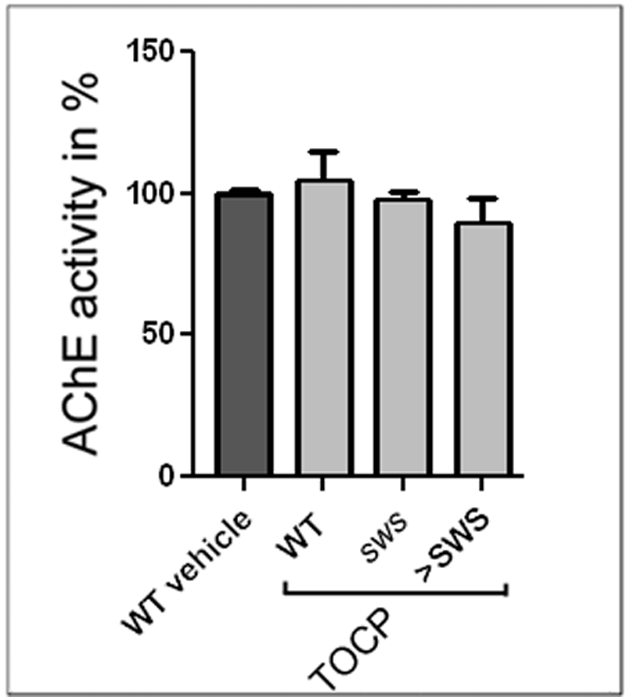

Supplement: Figure S1 — AChE activity after treatment with 32 mg/ml TOCP. Neither TOCP treatment nor SWS levels have a significant effect on AChE activity. Two independent measurements were performed for each genotype and treatment. Student's t-test was used to compare each treated group to its corresponding untreated one. SEMs are indicated. (The variances were not significantly different between treated and untreated flies for each genotype). (TIF) [file pone.0087526.s001.tif]

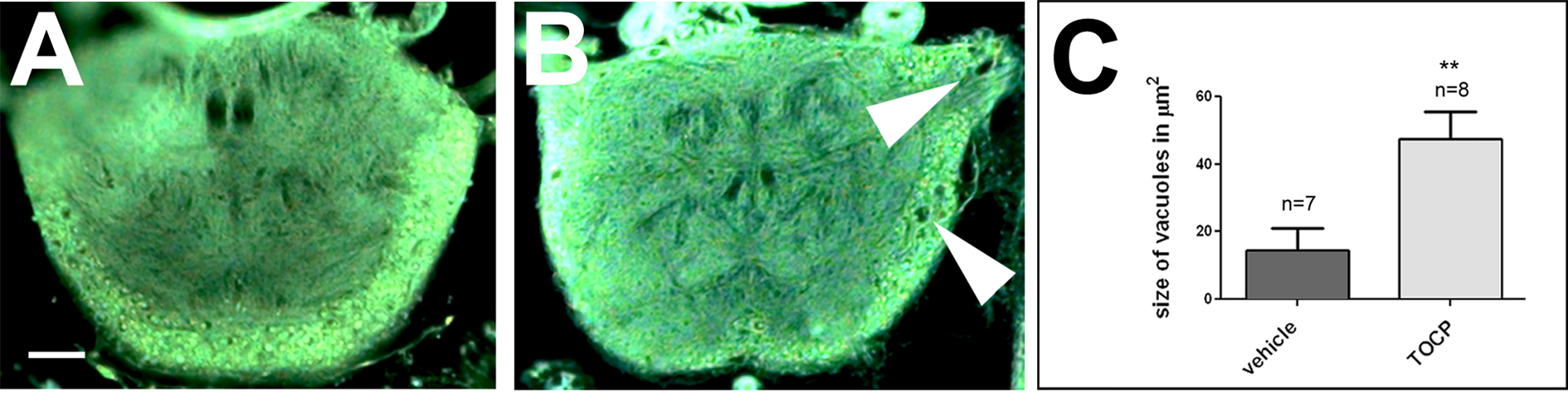

Supplement: Figure S2 — TOCP treatment results in vacuole formation in the thoracic ganglia. A. A paraffin section of a thoracic ganglia of a 14 d old wild type fly does not show vacuole formation whereas some vacuoles can be detected in a TOCP (32 mg/ml) treated fly (B). C. Measuring the area of vacuoles in the thoracic ganglia revealed a significant increase in TOCP treated flies. SEMs are indicated, n = number of thoracic ganglia analyzed; **p<0.01. Scale bar in in A = 15 µm. (There was no difference in variance). (TIF) [file pone.0087526.s002.tif]

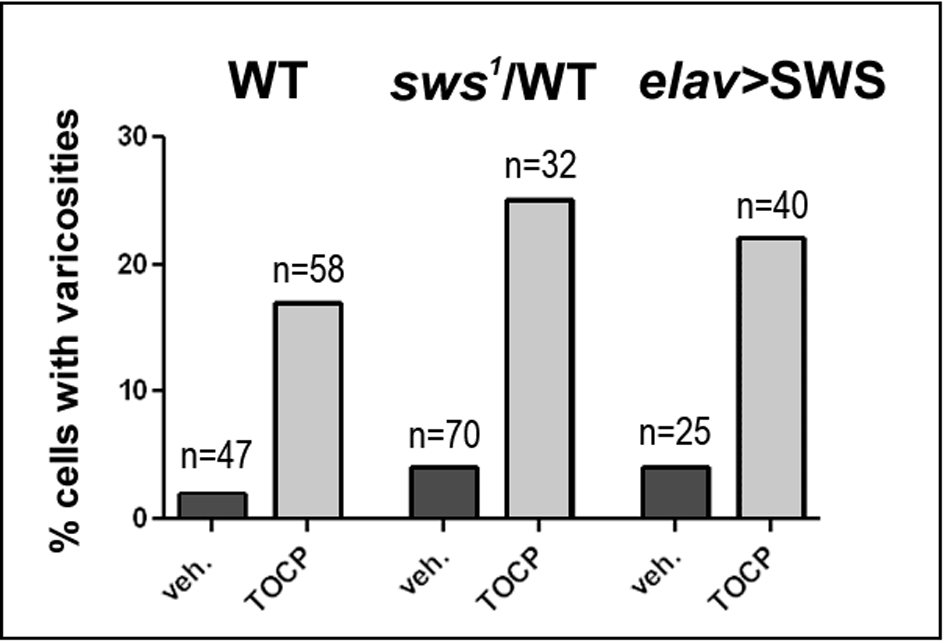

Supplement: Figure S3 — Varicosities are forming in cells treated with TOCP. Counting the percentage of cells that have developed varicosities reveals that substantially more cells show this sign of Wallerian degeneration when treated with TOCP (14 µg/ml). n = number of cells analyzed. (TIF) [file pone.0087526.s003.tif]

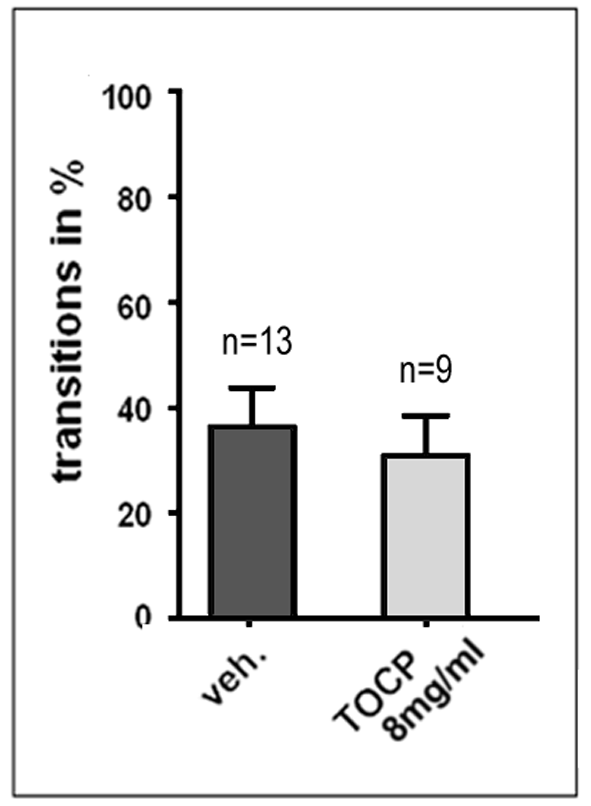

Supplement: Figure S4 — TOCP treatment does not induce defects in RING assays. Comparing untreated and TOCP treated flies in a RING assay did not reveal a difference in performance. Analysis was done with a Student's t-test and the SEM is indicated. n = is number of groups tested with 10–20 flies each. (There was no significant difference in the variance). (TIF) [file pone.0087526.s004.tif]

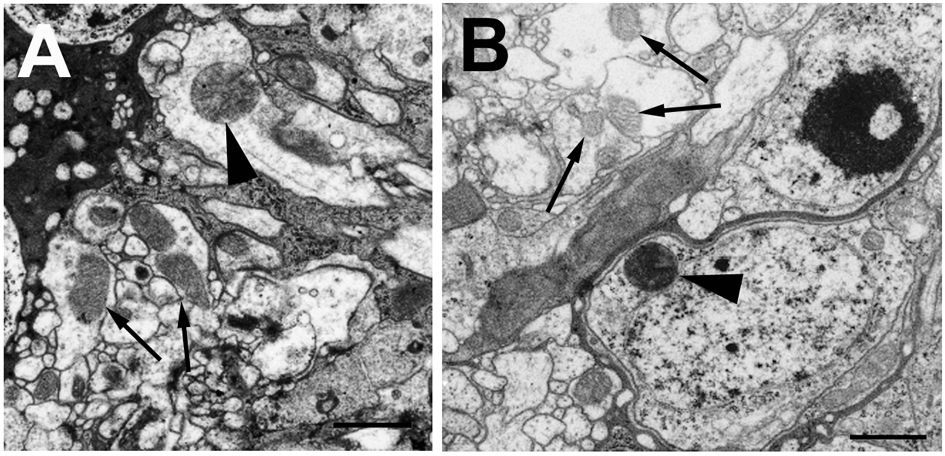

Supplement: Figure S5 — Altered mitochondria morphology in SWS overexpressing flies. A. A mitochondria in the axons shows abnormal cisternae and appears swollen (arrowhead) in comparison to normal looking mitochondria (arrows). B. Similar abnormal looking mitochondria can be found in neuronal cell bodies (arrowhead) whereas other mitochondria in the vicinity appear normal (arrows). Scale bar in A = 0.4 µm, in B = 0.8 µm. (TIF) [file pone.0087526.s005.tif]

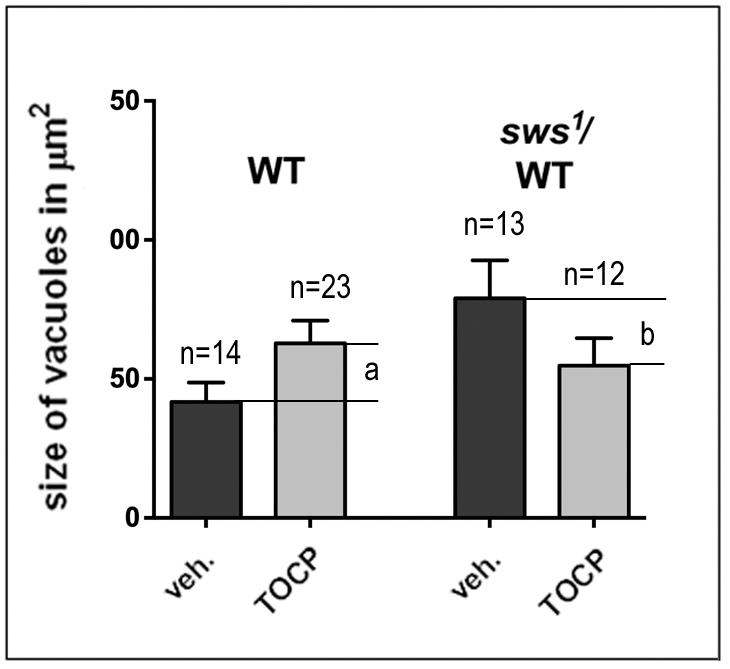

Supplement: Figure S6 — Interaction test to determine the effect of the genotype on the sensitivity to TOCP. Due to the increased vacuolization a generalized linear model following a gamma distribution (log-link) was used to compare the changes in the difference between treated and untreated wild type flies (a) and treated and untreated sws1 heterozygote flies (b). Only flies that showed vacuoles were used and the difference in the area of vacuoles was compared. This model revealed a significant difference with p = 0.018. n = number of sections analyzed. (TIF) [file pone.0087526.s006.tif]

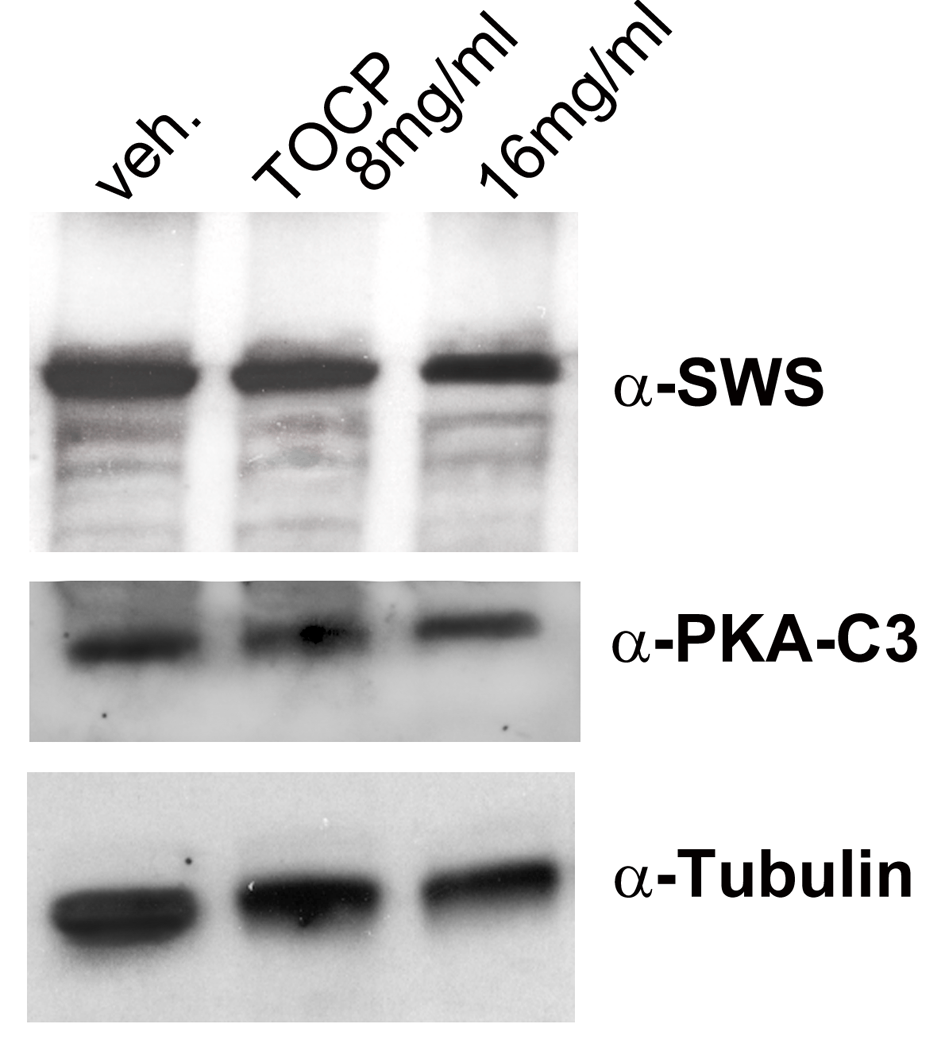

Supplement: Figure S7 — PKA-C3 and SWS protein levels are not affected by TOCP. Neither treatment with 8 mg/ml or 16 mg/ml did reduce the amount of SWS or PKA-C3. A loading control using anti-Tubulin is shown below. (TIF) [file pone.0087526.s007.tif]

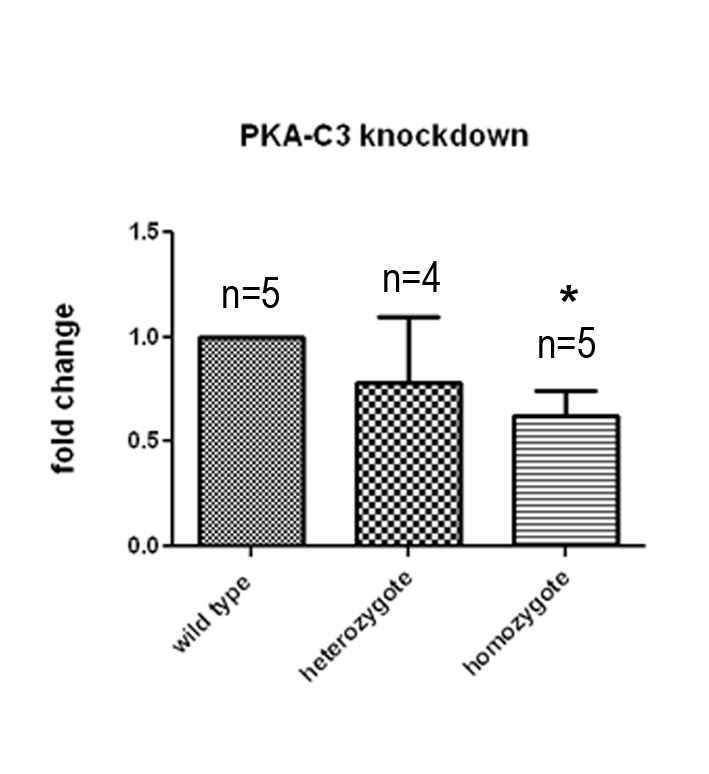

Supplement: Figure S8 — Fold change in PKA-C3 mRNA in the knockdown. Performing quantitative PCR we found a decrease in PKA-C3 mRNA levels in the heterozygous knockdown however this did not reach statistical significance. In contrast, the mRNA levels are significantly reduced in the homozygous knockdown. Triplicates of each genotype were used in each qPCR. Primers for actin were used as controls. SEMs are indicated, n = number of independent PCR reactions; *p<0.05. (TIF) [file pone.0087526.s008.tif]
